# The geography of COVID-19 in Sweden

**DOI:** 10.1007/s00168-021-01071-0

**Published:** 2021-07-23

**Authors:** Richard Florida, Charlotta Mellander

**Affiliations:** 1 Rotman School of Management and School of Cities, University of Toronto, Toronto, Canada; 2 Jönköping University, Jönköping, Sweden

**Keywords:** I10, J19, R23

## Abstract

This paper examines the geographic factors that are associated with the spread of COVID-19 during the first wave in Sweden. We focus particularly on the role of place-based factors versus factors associated with the spread or diffusion of COVID-19 across places. Sweden is a useful case study to examine the interplay of these factors because it did not impose mandatory lockdowns and because there were essentially no regional differences in the pandemic policies or strategies during the first wave of COVID-19. We examine the role of place-based factors like density, age structures and different socioeconomic factors on the geographic variation of COVID-19 cases and on deaths, across both municipalities and neighborhoods. Our findings show that factors associated with diffusion matter more than place-based factors in the geographic incidence of COVID-19 in Sweden. The most significant factor of all is proximity to places with higher levels of infections. COVID-19 is also higher in places that were hit earliest in the outbreak. Of place-based factors, the geographic variation in COVID-19 is most significantly related to the presence of high-risk nursing homes, and only modestly associated with factors like density, population size, income and other socioeconomic characteristics of places.

## Introduction

Why does COVID-19 hit some places harder than others? What are the characteristics of some places that leave them more vulnerable to the virus? What factors influence and shape the geographic variation and diffusion in COVID-19 across places? These are big questions that a growing body of research has begun to grapple with, and which are the focus of this study. The particular focus of this study is on distinguishing the role of place-based factors which are associated with the locational variation in COVID-19 but also factors associated with the diffusion of COVID-19 across place.

The lion’s share of the research to date has focused on the role of place-based factors in the geography of COVID-19. Initially, it was speculated that population size and especially density played a central role in the geography of COVID-19. This is likely because the earliest wave of the pandemic hit hardest at large global cities like New York and London. But a number of studies since have debunked this claim (Nathan 2020; Florida et al. 2020). Other studies find factors such as overcrowding, household size, transit use, race, poverty and inequality to be associated with the geographic variation of COVID-19 (Credit 2020; Drefahl et al. 2020). A number of studies have also focused on the geographic factors that are associated with the diffusion or spread of COVID-19 across places. While large cities were hit first and hardest, over time COVID-19 spread across various types of places, with significant incidence of both cases and deaths in smaller cities, suburban areas and rural places (Carozzi et al. 2020). Several studies trace the role of super-spreader events and tourist movements in the spread of COVID-19 across places (Kuebart and Stabler 2020; Chin and Bouffanais 2020).

In this paper, we examine the geography of COVID-19 in Sweden. We focus on the role of diffusion factors as well as place-based characteristics in the spread of the virus over the entire first wave of the pandemic in Sweden, roughly February to early August 2020. Sweden is a good study to examine the role of these two classes of factors because it did not impose mandatory lockdowns during the first wave meaning there were essentially no regional differences in the pandemic policies that could have affected the geographic variation in the incidence of COVID-19 in Sweden.

We find diffusion factors to be more important in understanding the geographic of COVID-19 than place-based factors. The most significant factor of all—proximity to places with higher levels of infections—is related to diffusion of the virus. COVID-19 is also higher in places that were hit earliest in the outbreak. The most significant place-based factor is the presence of high-risk nursing homes. The geographic variation in COVID-19 is only modestly associated with other place-based factors like density, population size and the socioeconomic characteristics of places. Ultimately, the place-based socioeconomic variables explain little of the geographic variation in COVID-19 across Sweden. When it comes to place-based characteristics, there appears to be a high degree of randomness in the geographic variation of COVID-19 across Sweden.

The remainder of the paper proceeds as follows. The next section introduces the conceptual and theoretical foundations of our analysis. The third section describes the variables, data and methodology used in the analysis. The fourth section provides background on the geography of COVID-19 in Sweden during the first wave of the pandemic. The fifth section summarizes the findings in our analysis of COVID-19 cases and deaths across Swedish municipalities. The sixth section summarizes the results for the analysis of COVID-19 cases across neighborhoods within cities. The concluding section highlights the key findings and discusses their relationship to prior research and what we know about the geography of COVID-19 generally.

## Theoretical foundations

Much previous research on the geography of COVID-19 has focused on the role of place-based factors like density, population, overcrowding, inequality, or socioeconomic disadvantage. Early in the pandemic, media reports suggested that COVID-19 was associated with larger denser places, likely because the virus hits first and hardest at large global cities like New York and London. But a range of studies have since debunked this claim (Nathan, 2020; Florida et al. 2020), finding mixed evidence on the role of density, with some studies showing only a modest positive association and others finding no association at all. A study of US metropolitan counties found COVID-19 cases and deaths to be associated with population size but not density; that county density is not significantly related to COVID-19 cases; and that higher density counties have lower death rates from COVID-19 (Hamidi et al. 2020). A study of US metro regions (Carozzi et al 2020) also found density to be associated only with the earliest outbreaks of COVID-19, but not associated with COVID-19 cases or deaths on a time-adjusted basis. In other words, large cities got hit first but not necessarily harder than smaller places over the long run. This is likely because large cities are more interconnected to other places across the globe through travel, tourism and flows of immigrants, but they tend to benefit from better health systems and are better able to implement physical distancing measures that help mitigate and contain the virus (Hamidi et al. 2020, Carozzi et al 2020).

Other studies find additional factors such as overcrowding, race, income, age and transit use to be associated with the geographic spread of COVID-19. In a study of the UK, Nathan (2020) finds COVID-19 cases to be associated with household size and public transportation usage. More importantly, he finds COVID-19 cases to be positively associated with socioeconomic deprivation. This is supported by numerous studies which document the disproportionate impact of COVID-19 on less advantaged groups and communities, racial and ethnic minorities, and frontline workers (Nguyen et al. 2020). Drefahl et al. (2020) document the disproportionate impact of COVID-19 on the less educated, those with lower incomes, immigrants and disadvantaged and vulnerable members of society, Another study (Credit 2020) documents the disproportionate impact of COVID-19 on neighborhoods with larger concentrations of Hispanic and Black populations, likely due to the combination of overcrowding of more multigenerational households and but also due to that many in these groups have more exposed jobs. The combination of overcrowding and socioeconomic deprivation is particularly important since a large body of research documents the role of household transmission in the spread of the virus (Lie et al. 2020; Bi et al. 2020).

In addition to these place-based factors, there are also factors that affect the diffusion of COVID-19 across places. There is a large literature in geography on spatial diffusion which notes the role of factors like information, knowledge and innovation in the diffusion of various kinds of activities (see Hägerstrand 1966, 1970; Audretsch and Feldman 1996; Anselin et al. 1997; Feldman 1999; Porter 2000)**.** Hierarchical models have been used to explain the spatial diffusion of infections (Hägerstrand 1967; Viboud et al. 2006). Diffusion is essentially the spread of things or activities across place, so two factors—proximity and connectivity—will matter significantly. It has long been known that movements of people and tourists are associated with the spread of infectious diseases (), and this is why border closures and restrictions are imposed to mitigate transmission (Linka et al. 2020). In the case of COVID-19, studies show that that super-spreader events and super-susceptible locations play a considerable role in its spread (Chin and Bouffanais 2020; Kuebart and Stabler 2020). Our research looks at the interplay of these two sets of factors—place-based factors and diffusion factors—in the spread of COVID-19 in Sweden.

## Variables, data and methodology

The data on COVID-19 cases are based on weekly data released by the Public Health Agency of Sweden (Swe: Folkhälsomyndigheten) on the number of confirmed infections. The data cover the period February 3, 2020, to August 2, 2020, which is the 25-week time period with the most COVID-19 cases during the first pandemic wave in the country.

The data on COVID-19 deaths come from the National Board of Health and Welfare (Swe: Socialstyrelsen). The definition of a COVID-19 related death in Sweden is if (1) a person is reported dead by a medical doctor via SmiNet (the database system used to report by law notifiable infectious diseases) or by the health care sector via the regional infectious restraints, or if (2) a person dies within 30 days after a confirmed COVID-19 test has been taken. The data cover all deaths up until the first week of August by municipality; however, at the neighborhood level, the data on deaths are not available on a weekly basis but is known for the entire period.

The analysis covers two levels of geography. The first is the municipal level. Swedish municipalities are similar to US counties and cover jurisdictional units that responsible for public services such as schools, emergency services and more general physical planning. Sweden consists of 290 municipalities, which together cover all its geographic area.

The second level of is the neighborhood level. This analysis covers 34 neighborhoods in Sweden’s three largest cities—Stockholm, Gothenburg and Malmö, neighborhoods for which data on COVID-19 cases is available from the Swedish Health Agency. However, the agency does not disclose the exact number of COVID-19 cases if the total number of infections (over time) is below 15. In these instances, we extrapolate the value s and add a dummy variable to control for the weeks where these data are not available. This primarily covers the earliest weeks of the pandemic when mainly smaller municipalities reported a limited number of cases.

The analysis combines these data on COVID-19 cases and deaths with data on a range of explanatory variables identified by the previous research as possible factors related to geographic spread and outbreaks. Most of the data for these variables come from Statistics Sweden, unless otherwise noted below.

### Dependent variables

#### COVID-19 Cases

The data on COVID-19 cases come from the Public Health Agency of Sweden and are the cumulative number of reported of infections They are reported on a weekly basis over the 25 weeks and are available for all Swedish municipalities and 34 neighborhoods in Stockholm, Gothenburg or Malmö. We examine cases on a per capita basis. When using this cumulative value, a dummy variable for week of the observation is added, since values by definition will be higher during later stages of the first pandemic wave.

#### COVID-19 deaths

These data on COVID-19 deaths come from the National Board of Health and Welfare and are the total number of COVID-19 deaths per municipality. We use total reported deaths per capita.

The data for COVID-19 cases and deaths are available on a daily basis at the national level. Data at the municipal and neighborhood level are more limited. The number of cases is available at a weekly level, but the number of deaths is only available for the overall time period.

### Independent variables

#### Place-based factors

#### Population size

Previous studies find an association between population size and COVID-19 cases and deaths. This variable captures the population size of each area.

#### Density

There has been considerable debate over the role of density in the spread of COVID-19. We measure density as people per square kilometer.

#### Age

At an individual level, age has been found to be a key factor in COVID-19 severity and death. Approximately 90 percent of those who died from COVID-19 in Sweden have been 70 years or older. However, older people have also been more isolated during the pandemic and therefore we would expect places with a larger share of young individuals to transmit the virus more rapidly.

#### Income

Income has also been found to be associated with COVID-19, with lower-income people and places being more vulnerable to the virus. We employ two different measures of income to capture both absolute and relative income: disposable 1000 SEK income per person, which includes wage income, transfers and capital income, and relative income which is the average income in the neighborhood (or municipality) divided by the average income in the municipality (or nation).

#### Income inequality

Income inequality has been found to be positively associated with COVID-19. Our variable is based on the Gini coefficient for disposable income.

#### Household size

Several studies have found that COVID-19 transmits more easily indoors when people are close to one another. We include two variables to household characteristics: the average number of individuals in the households and the share of single (one-person) households.

#### Multigenerational households

COVID-19 has been found to spread faster in multigenerational households. Our variable captures the share of households that includes both older (older than 70 years of age) and younger (under 15 years age) individuals.

#### Immigration status

Research has found places with higher shares of immigrants to be more vulnerable to the virus. This may be because they are characterized by greater overcrowding and higher rates of multigenerational households, have lower incomes, work in more frontline occupations, or lack language skills to understand the information from health authorities. For example, early reports from late March suggested that a reason the Somali community was hard hit had was due to a lack of public health information available in the Somali language (Swedish Television, 2020a). We employ two variables to capture immigration status: the share of the population born outside of Sweden and the share of the individuals who are native born but with one or two parents who are foreign born.

#### Education

Education has been shown to be associated with COVID-19. At the individual level, the relationship is expected to be negative, but at this aggregated regional level, we believe that education partly becomes a proxy for city size, since bigger cities have higher shares of educated and could be positively related to infections and death. It could also partly capture the ability to work remotely, given that many knowledge jobs have allowed for this. We include a variable for the share of the labor force with a BA or above.

#### Frontline occupation

Frontline occupation has been shown to affect vulnerability to COVID-19, with frontline workers being far more likely to be exposed to the virus. We thus include a variable for the share of frontline workers based on (1) the degree to which workers interact directly with the public and (2) jobs that require high levels of very close physical proximity to others (as per Florida. 2020). This is based on their place of residence, not place of work.

#### Unemployment

We include a variable for the share of the individuals aged 20–64 who are unemployed. The assumption is that those who are unemployed are less likely to interact with others, since they do have a workplace to go to, and that regions with larger shares of unemployed individuals also would have lower infection rates.

#### Nursing homes

In Sweden, as in many parts of the world, a disproportionate number of COVID-19 deaths occurred in nursing homes, among older people with pre-existing health conditions or comorbidities. Since approximately 70 percent of all deaths in nursing homes were in only 40 municipalities, IVO, the Health and Social Care Inspectorate conducted an evaluation of more than 1700 nursing homes’ routines across the country during the pandemic. We employ a dummy variable if the municipality is one of those on IVO’s list (Health and Social Care Inspectorate, 2020a. 2020b; Swedish Television, 2020b, c). Causality may not be clear in this case, since a municipality with many death cases may be more likely to be listed among the 40 municipalities on the IVO report list.

#### Diffusion-related factors

##### Week of first infection

Diffusion occurs in stages. Research documents that places that got hit first by the virus also got hit harder. We include a variable to capture the first week with a registered infection case in that neighborhood or municipality. The data come from the Public Health Agency of Sweden.

##### Proximity

Proximity is a key factor in diffusion processes, with diffusion more likely in adjacent places. We construct a spatial variable based on the distance to infections per capita in other regions weighted with the time it takes to go there (for the accessibility calculation methodology, see Appendix 1). We employ this variable both with a one- and two-week lag (for infection rates) and a two-week lag (for death rates, since we would expect an extra week to see an effect in death rates).

##### Air connectivity

Diffusion is related to connectivity as well. Previous research finds that more connected places have been hit earlier and harder by the virus. We include a variable for airport access based on the distance to the nearest airport, the time it takes to go there in combination with the number of passengers trafficking the airport (for the accessibility calculation we use the same methodology as for the spatial dependence variable, but instead use access to airport passengers). The data come from the Swedish Transport Agency (Swe: Transportstyrelsen).

We conduct both a correlation and a regression analysis of COVID-19 cases and deaths. We perform cross section regressions since only the dependent variable varies over time, while the explanatory variables reflect annual data that are not possible to estimate in a panel FE framework.

## Background of COVID-19 in Sweden

Sweden developed a unique response to the COVID-19 crisis, over which there has been considerable controversy (see, e.g., Karlsten 2020). In contrast to most other countries, Sweden did not impose a mandatory lockdown at neither the national nor regional level. Based on guidance from its public health authorities, the country implemented a “trust-based” strategy for staying open while protecting the old and vulnerable. The policy left schools, childcare centers, restaurants, bars and retail shops open, while encouraging workers and citizens to take proper precautions. The policy depended on information and trust to guide its response to COVID-19. People were advised to physically distance by keeping six feet apart rather than wearing face masks, to avoid crowds with more than fifty people and to wash their hands frequently. Companies transitioned to remote work, with primarily professional and knowledge workers working from home. Young children were allowed to attend day-care and elementary school, but high schools and universities were closed with classes conducted remotely. Gyms were closed temporarily, and travel across regions was discouraged. The strategy was far from perfect. Sweden suffered from a considerably higher fatality rate than its Scandinavian neighbors, with a disproportionate share of deaths occurring in its nursing homes.

Despite the fact that the Swedish strategy has been presented—especially in the international media (e.g., Goodman 2020)—as it had the goal of achieving herd immunity, it has never been stated to be the goal of Swedish expert authorities (Paterlini 2020). Rather, the focus has been on creating a strategy that as many as possible can live with for as long as possible, given that the pandemic was expected to last for a long time. The efficacy of the Swedish approach is beyond the scope of this paper and awaits further research. What is relevant to our study is that because the country did not implement a lockdown, Sweden provides a useful case to study geographic variation in the spread of COVID-19, since the country remained more open than most other countries. Further, there were essentially no regional differences in the pandemic mitigation policy or strategy during this time period that could have had an effect on the spread of the virus.

The official COVID-19 data in Sweden start on the February 4, 2020 (Week 6 in year 2020), and this date forms the starting point of our analysis. The COVID-19 crisis reached separate peaks for cases versus deaths. In terms of death rate, the crisis peaked in Weeks 15 and 16 (beginning of April). In terms of reported cases, the peak came in June (Weeks 24 to 26). The case peak may be a result of more extensive testing across a larger population group. Initially, primarily individuals at risk and in the health care sector got access to COVID-19 infection tests. Testing then became more available later, which may generate some bias in the case numbers.

## Geographic variation in COVID-19 by municipality

**Fig. 1:**
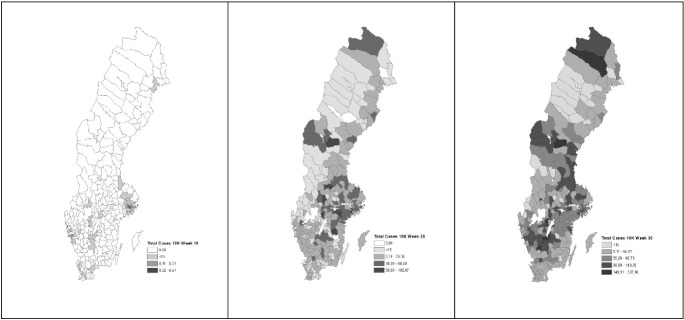
The Geography of COVID-19 Cases per Capita for Swedish Municipalities. Note COVID-19 Cases per 10,000 people as of Weeks 10, 20 and 30

We now move on to our analysis of the geographic variation in COVID-19 across Swedish municipalities. The geography of COVID-19 cases is highly uneven across Sweden. Fig. 1 maps infections on a per capita basis. These data are available on a weekly basis from the start of the pandemic. We map three points in time: Week 10 (first week of March), Week 20 (second week of May) and Week 30 (third week of July) to show the geographic spread of cases.

The geographic pattern of COVID-19 in Sweden changed somewhat over time. Following a pattern seen in other nations, the crisis hit larger municipalities first and then spread to smaller places across the country. Up to Week 10, larger municipalities like Stockholm, Gothenburg and Malmö bore the disproportionate bulk of COVID-19 cases. By Weeks 20 and 30, the pattern had partly shifted to smaller municipalities. The correlation between cases per capita and population size was 0.23** in Week 10, 0.20** in Week 2 and 0.20** in Week 30.

### Correlation results

Table 1 provides the results of a partial correlation analysis for COVID-19 cases per capita on a weekly basis (from Week 6 to Week 32) controlling for week and a bivariate correlation analysis for COVID-19 deaths per capita by Week 32 (the first week of August).

**Table 1:** Correlation Analysis for COVID-19 in Swedish municipalities

	Partial correlation	Bivariate correlation
	Cases per capita	Deaths per capita
Population (ln)	0.151**	0.121*
Density (ln)	0.132**	0.192**
Average age	−0.171**	−0.122*
Over 70 years of age	−0.179**	−0.128*
Income	0.073**	0.061
Income inequality	0.080**	0.074
Average household size	0.124**	0.093
Intergenerational households	0.075**	0.045
Single households	−0.020	0.074
Foreign born	0.131**	0.228**
Second-generation immigrants	0.170**	0.214**
Education (BA and above)	0.085**	0.019
Frontline occupations	−0.138**	−0.093
Unemployment	−0.152**	0.003
Average education in nursing homes	–	−0.106
Nursing home problems IVO	–	0.588**
Air connectivity	0.132**	0.265**
Week of first case	−0.198**	−0.152**
1 Week lag infection rates own municipality		0.578**
1 Week lag access. to total cases per cap in other regions	0.589**	–
2 Week lag access. to total cases per Cap in other regions	0.573**	0.296**

* indicates significance at the 1 percent level

** indicates significance at the 5 percent level

Partial correlation controlling for week of the pandemic. Cases per capita are based on weekly data. Deaths per capita are based on total deaths by the first week of August

The variables for population size and density are positively associated with COVID-19 cases and deaths. Those results are partly skewed by the Stockholm region, which was hit hard early in the pandemic. The results also show that the average number of individuals per household is positively and significantly associated with COVID-19 cases. There is also a positive and significant association between the variable for multigenerational households and COVID-19, though neither of these variables is strongly associated with COVID-19 deaths. This is partly in line with other research which finds multigenerational households to be factors in COVID-19 transmission (Hamidi, 2020). There is no significant association between the share of single households and COVID-19 cases and deaths.

The coefficients for age-related variable correlations are negative and significant. Most likely, older people have been more isolated during the pandemic and therefor also been less likely to transmit the virus. Even though risks of becoming seriously ill or die increase with age, at this aggregated level, “average age” is more likely to capture the share of the population that still have been in contact with others and with that more likely to spread the virus.

We now turn to the variables for income, education and frontline occupation. Prior studies note that COVID-19 cases and deaths have disproportionately concentrated among less advantaged groups and communities and frontline workers who have more frequent contact with other workers and the general public. In Sweden, for example, the most vulnerable occupations are taxi drivers, bus drivers and pizza makers with a risk 4 to 5 times greater that of the average worker (Swedish Public Health Agency, 2020). Our findings for these variables are counter-intuitive. The variables for income and education are both positively and significantly associated for COVID-19 cases. Even more counter-intuitively, there is a negative and significant association between the variable for frontline workers and COVID-19 cases. These results may be artifacts of a “big-city” effect, where larger places, which are also more affluent and educated, were harder hit by the virus. This is also suggested by the correlation for income inequality and COVID-19 cases which is positive and significant. Here, we note that the data are municipal averages and as such do not account for potentially significant within-municipality differences of neighborhood levels of income or education—a subject we will cover in the neighborhood-level analysis. The correlations for both income and income inequality are insignificant with COVID-19 deaths. Unemployment is negative and significant with COVID-19 cases. This may reflect that fact that Sweden remained open with more workers continuing to go to work, which may have resulted in more employed workers contracting the virus wherever unemployment levels are low.

In many nations across the world, a significant share of COVID-19 deaths has been concentrated in nursing homes. Roughly three-quarters (73 percent) of COVID-19 deaths in Sweden have been among individuals who either have lived in nursing homes (47 percent) or who needed special assistance in their own homes (26.5 percent). The variable for the presence of high-risk nursing homes (based on 40 municipalities that accounted for the largest share of nursing home deaths) is positive and significantly associated with both cases and deaths.

The strongest correlation, when it comes to infection rates per capita, is the access to other regions with higher levels of infection rates per capita. This suggests that municipalities that have been located close to other regions with high infection rates one and two weeks earlier, also experienced higher infection rates later on. The spatial dependence is stronger for infection rates than for deaths, but positive and significant in both cases. In the case of deaths, high numbers of infections in the own municipality 1 and 2 weeks earlier are positive and significant.

We also find a positive and significant correlation between places that got their first infections during the first weeks of the pandemic and overall COVID-19 cases and deaths.

The coefficient for air connectivity is also positive and significant, suggesting that the connectivity of large places may have played a role in their being hit earlier and harder by the pandemic. This is line with the findings of Carozzi et al. (2020).

### Regression findings

We now turn to the findings of our regression analysis for Swedish municipalities. Recall municipalities in Sweden include cities and their outlying urban areas. The regression analysis enables us to better deal with the fact that many of the variables in the analysis essentially reflect and capture similar underlying elements of city-regions. The analysis excludes explanatory variables that are insignificant in the correlation analysis. Since many of them are related to population size which may result in multicollinearity problems, we first run a principal component analysis to extract a “big city variable.” The result of the principal component analysis is available in Appendix (1). We include the significant variables from the correlation analysis, that can be expected to be related to regional size and we create a component based on the covariances. This component (see component 1, Table A in Appendix) is strongly related to population size and density, incomes, age structures, air connectivity, educational and occupational structures, and household size. We employ this component in the first regression as a proxy for “big city characteristics.” Since we based the component on covariances and not correlations, some included variables may get a stronger weight into the component used in the analysis (Jolliffe 1986). We report both the unstandardized/standardized β-coefficients to shed light on the relative importance of the variables. Since only the dependent variable varies over time, while the explanatory variables are yearly data, it is not possible to estimate in a panel with fixed effects. The data are constructed in a panel framework with a traditional OLS estimation where we control for the week of the pandemic. Column 1 provides the estimations for cases per capita, based on the variables that were significant in the correlation analysis (Table 2).

**Table 2:** Regression analysis for COVID-19 cases per capita

	1	2	3	4	5
*Big city component*	2.282**/0.071	2.287**/0.071	−0.099/−0.003	−1.674/**/−0.051	−1.611**/−0.049
*Nursing home*					
Nursing Home Problems IVO			17.730**/0.189	12.618**/0.134	13.534**/0.142
Week of First Case	−1.240**/−0.094	−1.236**/−0.094	−1.304**/−0.099	−1.113**/−0.084	−1.185**/−0.088
*Spatial lags*					
1 Week Lag Access. to Total Cases per Cap				7.166**/0.675	
2 Week Lag Access. to Total Cases per Cap					7.529**/0.644
Week dummy	No	Yes	Yes	Yes	Yes
N	7539	7539	7539	7249	6954
R2 Adj	0.021	0.521	0.551	0.695	0.680

** indicates significance at the 5 percent level

Cases are based on weekly data

Regression 1 estimates the role of “big city characteristics” while still controlling for the week when the municipality had the first COVID case. While it is positive and significant, the R2 Adj value shows that it in fact explains very little of the variation of infection rates per capita across municipalities. In regression 2, we add a week fixed effects variable which increases the R2 Adj to approximately 0.52. This is as expected as the number of cases increased over time. In regression 3, we add the variable that captures the municipality that was listed as problematic by IVO and that lacked proper routines to deal with the infection during the first wave. When adding this variable, the big city variable becomes insignificant, and the increase in the R2 Adj is very minor (from 0.531 to 0.551). In regression 4 and 5, we add two separate spatial lags that capture accessibility to infection rates per capita in other municipalities weighted by the time it takes to go there. Both lags are significant, with the 1-week lag being slightly stronger of the two. The R2 Adj increases to 0.680–0.695 which indicates that much of the variation across municipalities is not so much explained by their own characteristics but rather their geographic location relatively other affected regions. This indicates a certain randomness in how the virus has been spread and that it seems to have had relatively little to do with the socioeconomic characteristics of the municipalities. Worth noting is that the big city component also changes sign and becomes significant when combined with the spatial lags. The bivariate correlation between the big city component and the spatial lag is only approximately 0.12, which makes us believe that the negative sign is not due to multicollinearity problems in the model. When we run regression 3 and 4, the variance inflation factor (VIF) values are all below 1.6 which also confirms this. The exception is the VIF values for the spatial lag variables and the week dummy since cases are expected to increase also in other regions over time. Still, the VIF values are below 3.2. Next, we run a regression analysis but now with COVID-related deaths per capita as the dependent variable (Table 3):

**Table 3:** Regression analysis for COVID-19 deaths per capita

	1	2	3	4
*Big City Component*	4.350/0.100	−6.154*/−0.142	−6.396**/−0.147	−5.911**/−0.136
Nursing Home Problems IVO		78.030**/0.620	62.264**/0.495	62.394**/0.496
Infection Rates Own Municipality 1 Week Lag			0.485**/0.422	0.529**/0.481
Week of First Case	−1.709/−0.096	−2.011*/−0.113	−0.633/−0.036	−0.608/−0.034
*Spatial lags*				
2 Week Lag Access. to Total Cases per Cap				−0.994/−0.073
N	290	290	290	290
R2 Adj	0.023	0.360	0.523	0.524

* indicates significance at the 1 percent level

** indicates significance at the 5 percent level

Starting with regression 1 that includes the big city component in combination with the control for when the first case, we see that the big city component is insignificant. Neither do we find more deaths per capita in regions that experienced infection cases early on. The regression has an R2 Adj of only 0.023. In regression 2, we add the variable that captures if the municipality has been listed by IVO due to lacking routines in nursing homes during the first wave of the pandemic. This variable is, as expected, positive and significant. The R2 Adj increases to 0.360, which indicates that this explains approximately one-third of the variation across municipalities. In regression 3, we add a variable for the infection rates in the municipality the week before. This variable is also positive and significant, and approximately of the same magnitude as the nursing home problem variable (a standardized β-coefficient of 0.442). The VIF values are all below 1.6 in the regression which indicates that the changed sign of the “big city component” variable, which now is significant, is not because of multicollinearity problems. The R2 Adj is now 0.523 which indicates that approximately half of the variation in COVID-related deaths per capita can be explained by nursing home problems and overall infection rates in the municipality. When we add variables for the accessibility to other regions with a 2-weeks lag (regression 4) which turns out insignificant. Based on the results in Table 2, we would still expect an indirect relation, since the spread in other regions explains much of the variation in infection rates per capita across municipalities.

## Geographic variation in COVID-19 by neighborhood

COVID-19 not only varies across city-regions or municipalities; it varies considerably within them. Neighborhoods within cities may be sorted and segregated by income, education, age, family size and status, nationality and other factors. Prior research has found these factors to be associated with patterns of COVID-19 cases and deaths. To get at this, we examine COVID-19 cases across Swedish neighborhoods. (Data on COVID-19 deaths are not publicly available for Swedish neighborhoods). As noted above, these neighborhood data are available for COVID-19 cases on a weekly basis for Sweden’s three largest cities: Stockholm, Gothenburg and Malmö. These cities are home to nearly one-fifth (18.4 percent) of the total population of Sweden.

**Fig. 2:**
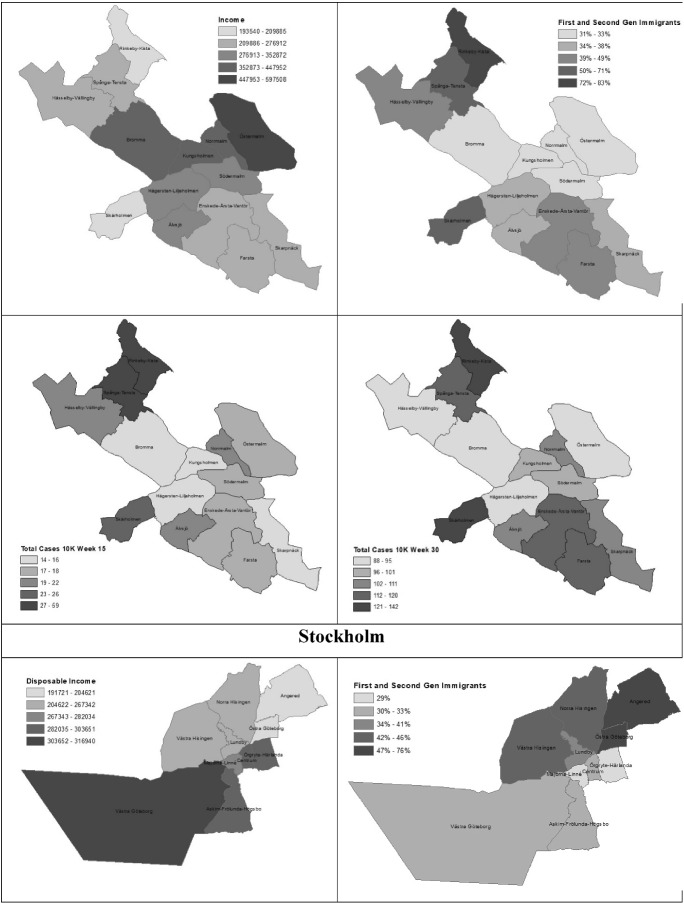

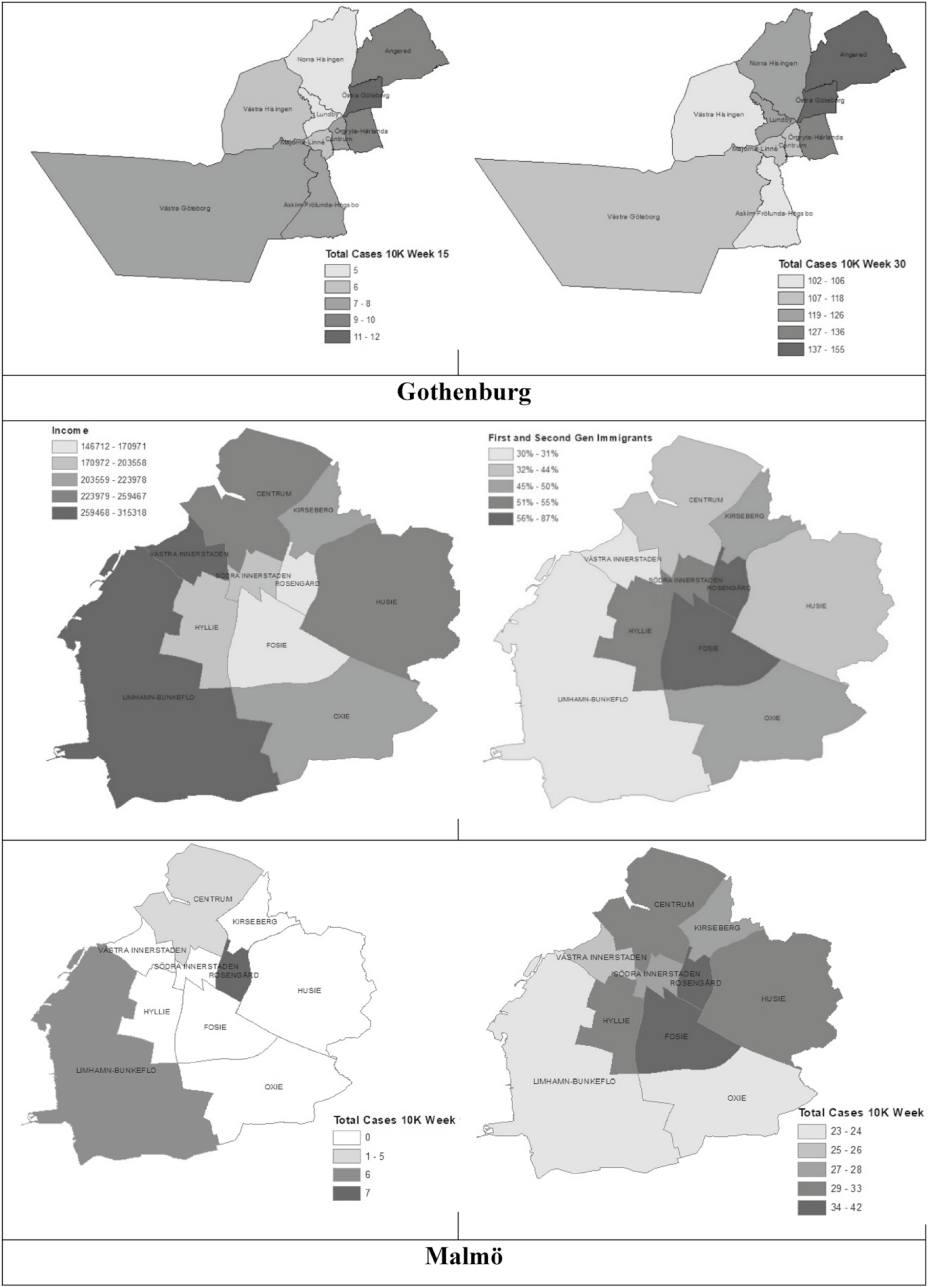
COVID-19 Cases across Neighborhoods in Stockholm, Gothenburg and Malmö. Note For Weeks 15 and 30

Figure 2 compares the concentration of COVID-19 cases at two time points in the pandemic (weeks 15 and 30) to the geographic patterning of income and immigrants in Stockholm, Gothenburg and Malmö, respectively.

In Stockholm, the neighborhoods with the lowest incomes and highest shares of immigrants (e.g., Rinkeby-Kista, Spånga-Tensta and Skärholmen) had higher concentrations of COVID-19 at both time periods.

A similar pattern is visible in Gothenburg, where COVID-19 cases are higher in neighborhoods with lower incomes and higher concentrations of immigrants (Angered, Östra Göteborg, but also Norra and Västra Hisingen), with the exception of one neighborhood, Västra Hisingen, where COVID-19 cases were relatively lower.

The results in Malmö follow a somewhat different pattern. In the initial stages of the pandemic, both high- and low-income neighborhoods were hit relatively hard, as can be seen in the examples of Limhamn-Bunkeflo which has high incomes and low shares of immigrants, and Rosengård, a disadvantaged and marginalized neighborhood. But by the later stages of the first pandemic wave, the geography of COVID-19 in Malmö is more similar to that of Stockholm and Gothenburg where COVID-19 is disproportionately concentrated in less advantaged neighborhoods.

To better understand the geography of COVID-19 at the neighborhood level, we turn to the findings of our correlation and regression analyses.

### Correlation results

Table 4 reports the results of a partial correlation for COVID-19 cases. It covers per capita cases on a weekly basis, controlling for the week data was registered and the municipality. The variables are similar to the analysis for municipalities, with the following exceptions. We use a variable for relative income that compares neighborhood income to city income to capture income inequality. We also exclude the air connectivity variable, since this will not vary for neighborhoods within cities. Neither do we have the report about nursing home from IVO by neighborhood but only by municipality and the variable will therefore be excluded.

**Table 4:** Results of neighborhood-level correlation analysis

	Partial correlation
	COVID-19 cases per Capita
Population (ln)	0.188**
Population density (ln)	−0.134**
Average age	−0.149**
Over age 70	−0.144**
Income	−0.076*
Income inequality (Relative Income)	−0.212**
Average household size	0.221**
Multigenerational households	0.244**
Single households	−0.030
Foreign born	0.160**
Second-generation immigrants	0.141**
Education (BA and above)	−0.121**
Frontline occupations	0.209**
Unemployment	−0.075*
Week of first case	−0.315**
Spatial lags:	
2 Week lag access. to total cases per cap	0.876**

* indicates significance at the 1 percent level

** indicates significance at the 5 percent level

Based on weekly data and controlling for week during the pandemic

First off, population size at the neighborhood level remains positively and significantly related to COVID-19 cases, but we now see the correlation coefficient for density is negative and significant. This might suggest that density is not necessarily a factor in the spread of COVID-19 cases across neighborhoods within cities.

COVID-19 cases at the neighborhood level are positively and significantly related to our variable for average household size and for multigenerational households, in line with prior research.

The findings for income, education and frontline occupation differ from the municipal analysis and more in line with prior research. The variable for income is negative and significant, indicating that COVID-19 cases are concentrated in the less advantaged neighborhoods. In addition, the coefficient for our measure of inequality (relative income) is even stronger, highlighting the connection between lower relative income and COVID-19 cases. The variable for educational attainment is also negative and significant. In contrast to the municipal-level results, the variable for frontline workers is positive and significant, again in line with prior research and intuitive expectations (Nguyen et al. 2020). The variables for frontline workers and educational attainment reflect place of residence, not place of work.

The result for immigration status is also positive and significant, in line with prior research. COVID-19 cases at the neighborhood level are negatively and significantly correlated with age. In other words, there are more COVID-19 cases in neighborhoods with a relatively younger population. This likely reflects the fact that the average age is lower in disadvantaged neighborhoods. It may also reflect the fact that Sweden remained relatively open, allowing younger people who are at lower risk to interact more with one another.

The variable for the weekly onset of first infection is also positive and significant. As with municipalities, timing matters: Neighborhoods that got hit first in general got hit harder.

By far the strongest association in this analysis is the variable that captures the spatial lag and infection rates in nearby municipalities two weeks earlier. This indicates that spatial dependence will explain a lot also of the neighborhood level variation in the three biggest municipalities. If the municipality is located nearby other municipalities with high infection rates per capita, the neighborhood levels per capita in Stockholm, Gothenburg and Malmö increased as well. This suggests that factors related to diffusion matter more than place-based characteristics in the spread of COVID-19 across places.

### Regression findings

We now turn to the findings for our neighborhood-level regressions. This analysis covers COVID-19 cases per week, from week 6 to week 32 in 34 neighborhoods in Stockholm, Gothenburg and Malmö. Given that some of the variables are closely related to one another, we again use a factor analysis to reduce the variables related to neighborhood characteristics. In this way, the factor analysis helps to reduce problems with multicollinearity. The generated components build on covariances and not correlations, which means that some included variables get a stronger weight into the component (Jolliffe 1986).

The results from the factor analysis, generate three separate components (the factor loadings are available in Appendix 3). Factor 1 reflects more disadvantaged neighborhoods—those with lower incomes, lower relative income, higher shares of first- and second-generation immigrants, lower educational attainment, more frontline workers, higher levels of unemployment, bigger households and higher levels of multigenerational households.

The other two factors mainly reflect the age structure of the neighborhoods. Factor 2 primarily reflects somewhat older neighborhoods, where the average age is higher and there is a larger share of the population aged 70 and above. These neighborhoods also have lower densities and lower shares of second-generation immigrants. Factor 3 primarily reflects somewhat richer neighborhoods, also with somewhat a higher share of second-generation immigrants and intergenerational households.

We use Factors 1 and 2 in the regression analysis relating neighborhood-level characteristics to COVID-19 cases. We add an additional regression to capture the timing of first infection (Regression 1). We also add a dummy variable to control for municipality. Table 5 summarizes the key findings from the regression analysis.

**Table 5:** Neighborhood-level regression analysis for infections per capita

	1	2	3
*Residential characteristics*			
Factor 1	6.892**/0.128 (0.974)	6.827**/0.127 (0.959)	10.070**/0.186 (0.443)
Factor 2	–	−5.002**/−0.093 (0.934)	−0.648/−0.012 (0.431)
Week of First Case	−4.159**/−1.819 (1.816)	−3.251/−0.038 (1.798)	−3.358**/−0.039 (0.824)
2 Week lag access. to total cases per cap	–	−	14.820**/0.909 (0.249)
Municipality dummy	Yes	Yes	Yes
Week dummy	Yes	Yes	Yes
N	917	917	883
R2 Adj	0.718	0.727	0.945

** indicates significance at the 5 percent level

Regression 1 finds a significant association for Factor 1. COVID-19 cases are thus associated with less advantaged neighborhoods, i.e., those with lower incomes, lower relative incomes (a measure of inequality), lower levels of education, more frontline workers and more first- and second-generation immigrants.

Regression 2, which adds a factor (Factor 2) to capture the age structure of neighborhoods. Factor 2 s negative and significant, but of lower magnitude than Factor 1 with a standardized β-coefficient is −0.093 compared to 0.127 for Factor 1. Here, it is important to remember that the analysis covers only COVID-19 cases not deaths. The Swedish strategy has been to have risk groups such as those in older age groups to remain isolated with limited contact with others. And because Sweden remained relatively open, young people were more likely to come into contact with the virus. The results from Regression 1 also suggest that neighborhoods that were hit early on had more infection cases during the pandemic, but this factor is not significant when adding Factor 2 to the regression.

In Regression 3, we add a spatial lag that captures the access to infection rates in nearby municipalities two weeks earlier. This variable is by far the strongest among the included in the regression, but Factor 1 remains significant. Factor 2, however, now becomes insignificant. This again reflects the role of diffusion factors in the spread of COVID-19.

Although place-based factors at the neighborhood level are significant, they explain relatively little of the variation of COVID-19. In fact, the dummy variables for Week and the Municipality Dummy explain substantially more of the variation, approximately 70 percent, compared to just 1 percent for the variables for neighborhood characteristics. Taken together, the results indicate that while neighborhood disadvantage is associated with COVID-19 cases, neighborhood characteristics do not explain much of the variation across these neighborhoods.

## Conclusion

This research has examined the role of place-based versus diffusion factors in the geographic incidence of COVID-19 in Sweden during the first wave of the COVID-19 pandemic from February to the beginning of August 2020. As noted earlier, Sweden provides a useful case study to examine the interplay of these two classes of factors in the geographic incidence and spread of COVID-19 since the country did not impose lockdowns and there was essentially no regional or place-based variation in COVID-19 mitigation policies or strategies during this time period.

Our findings suggest that diffusion factors are significantly more important than place-based factors in the spread of COVID-19 across places.

For one, proximity matters. The factor that explains the most variation in our models is proximity to other places with higher rates of infection. This factor explains more than half of the variation in COVID-19 across Swedish municipalities. And it is significant for in our models for neighborhoods within cities and as well municipalities.

Also significant is the timing of first infection. And again, this matters not just across municipalities both across neighborhoods within the three biggest municipalities as well. Swedish municipalities and neighborhoods that were hit earlier by the virus seem to have been hit harder—a finding which is in line with previous research.

The findings suggest that place-based factors play a far less significant role in the geographic incidence and spread of COVID-19 in Sweden.

We find density and population size, two factors that the media seized upon early in the pandemic, to be weakly correlated to the geographic variation in COVID-19 infections—both in municipalities and neighborhoods.

Our correlation findings suggest several factors to be equally or more important than either density or population. For example, household size is positive and significant in relation to infections at both the municipal and neighborhood levels. This finding is in line with prior research which identifies secondary transmission within households as a factor in the overall transmission of COVID-19.

We know that age is a risk factor at the individual level, with people over age 70 being much more likely to be hospitalized and to die from COVID-19. But at the municipal level, we find COVID-19 cases and deaths to be negatively associated with the variables for age. This likely reflects the fact that younger people were free to have contact with one another because Sweden did not impose any lockdowns, while older and more vulnerable people were more likely to isolate and physically distance.

The findings for the variables for socioeconomic status are mixed and somewhat counter-intuitive. The variables for income and education are positively related to COVID-19 cases at the municipal level, which seems counter-intuitive. However, as in other studies, the variable for income inequality is positively associated with COVID-19 cases across municipalities, as expected. These findings may reflect a more general result for city size, as bigger cities tend to be more affluent and educated but also more economically unequal. The factor for disadvantaged neighborhoods is modestly associated with COVID-19 cases in the neighborhood-level analysis.

The findings for frontline workers also generate mixed results at the municipal level but are positively and significantly related to COVID-19 cases in the neighborhood-level analysis. The variables for immigrants are positively and significantly associated with COVID-19 cases and deaths. This may provide additional evidence for the association between COVID-19 and disadvantaged neighborhoods, as immigrants in Sweden are generally concentrated in less advantaged neighborhoods.

The most significant place-based factor is the presence of high-risk nursing homes. Indeed, the presence of such homes is a stronger factor in COVID-19 deaths across municipalities than our component variable for big city characteristics.

When it comes to place-based factors, our findings suggest that the geographic variation of COVID-19 across Swedish municipalities is only weakly related to socioeconomic factors, and even fewer of these variables are significantly directly related to deaths from COVID-19. The neighborhood-level analysis finds the geographic variation in COVID-19 cases to be associated with the factor for neighborhood disadvantage. But again, this factor explains very little of the infection rates on a weekly basis during the pandemic.

The place-based factor that is most closely associated with COVID-19 across Swedish municipalities is the presence of at-risk nursing homes. This variable outperforms all others socioeconomic variables in the regression analysis for COVID-19 deaths across Swedish municipalities.

That said, place-based factors explain only a small amount of the geographic variation of COVID-19 across Sweden—in fact, roughly only ten percent of the variation in COVID-19 cases across municipalities and just one percent across neighborhoods. A couple of factors may bear on this. Differences in testing of COVID-19 across places may be skewing the data on cases. It may also be that the effect of variables that matter at the individual level may be muted when we aggregate up to the level of neighborhoods or municipality.

Perhaps the biggest takeaway from this study is that the geographic variation in COVID-19 and the vulnerability of certain places to it appear to have relatively little to do with their own characteristics. The spread of COVID-19 has much more to do with factors which bear on diffusion, particularly location near hard-hit regions. In a country that did not impose a lockdown or where there was little variation in regional policies to mitigate the COVID-19 pandemic, we find that it is not so much place-based characteristics that mattered to the spread of COVID-19 but rather location, randomness and bad luck.

These findings should be considered as interim results, in light of the caveats above. The pandemic remains ongoing, and its geographic pattern and determinants continue to evolve and are far from fixed. It is important to continue to track the geographic spread of COVID-19 and the factors associated with it. We encourage further research especially on the factors associated with the diffusion and spread of COVID-19 across cities and neighborhoods. Ultimately, achieving better understanding of the diffusion of COVID-19 is likely to evolve alongside the evolution of the virus itself.

## Appendix 1: Accessibility methodology

To estimate the accessibility infection rates in other regions we define it as follows: Each municipality (i) offers access to a Y, where

$$Y \in \left\{ {\text{No of Infections}} \right\}$$

But there is also an access to Y in the closely located municipalities (j). This is the case for all municipalities in the set N {1,…,n}. The total Y in municipality *i* is defined as follows:

$$A_{i}^{Y} = Y_{i} f\left( {c_{ii} } \right) + Y_{j} f\left( {c_{ij} } \right) + \ldots + Y_{n} f\left( {c_{in} } \right)$$

where $f(c)$ is a distance decay function to determining how the accessibility value is influenced by the related cost of reaching these infected individuals. Johansson et al. (2003) approximate this specific relationship by an exponential function:

$$f\left( {c_{ij} } \right) = \exp \left\{ { - \lambda t_{ij} } \right\}$$

where t_ij_ is the time distance between municipality i and j. λ is a time-sensitivity parameter that determines how the accessibility changes in t. λ has been estimated by Johansson et al. (2003) and reflects the implicit value of daily time use.

When we combine the two equations, we get the Y accessibility in municipality i is defined as follows:

$$A_{i}^{Y} = \mathop \sum \limits_{j = 1}^{290} Y_{j} \exp \left\{ { - \lambda t_{ij} } \right\}$$

In this analysis, we will thereby account for the infection rates that are accessible in other municipalities, but we discount the values of those by the time it would take to get there. In a similar manner, we calculate the accessibility to population, to be able to calculate accessibility measures per capita.

## Appendix 2

See Table

**Table 6:** Principal component analysis for municipalities

	Component			
	1	2	3	4
Population (ln)	0.728	0.084	−0.181	0.393
Density (ln)	0.865	0.114	0.022	0.224
Average age	−0.869	−0.113	0.404	0.069
Over 70 years of age	−0.863	−0.131	0.386	0.067
Income	0.724	−0.467	0.380	0.127
Income inequality	0.594	−0.132	0.679	0.261
Average household size	0.803	0.047	−0.049	−0.425
Intergenerational households	0.361	−0.149	0.453	−0.672
Foreign born	0.469	0.818	0.132	−0.025
Second-generation immigrants	0.680	0.601	0.093	−0.090
Education (BA and above)	0.795	−0.340	0.054	0.295
Frontline occupations	−0.799	0.196	0.026	0.071
Unemployment	−0.325	0.775	0.377	0.163
Air connectivity	0.867	−0.059	−0.035	−0.088

^6^.

## Appendix 3

See Table

**Table 7:** Principal component analysis for neighborhoods

	Component		
	1	2	3
Population (ln)	−0.606	−0.321	0.120
Density (ln)	−0.300	−0.749	0.067
Average age	−0.371	0.832	0.099
Over 70 years of age	−0.367	0.827	0.035
Income	−0.854	0.059	0.457
Income inequality	−0.847	0.261	0.157
Average household size	0.837	0.294	0.359
Intergenerational households	0.710	0.278	0.586
Foreign born	0.930	−0.119	0.099
Second-generation immigrants	0.506	−0.546	0.487
Education (BA and above)	−0.931	−0.283	0.089
Frontline Occupations	0.851	0.190	−0.301
Unemployment	0.819	−0.021	−0.062

^7^.

## References

[CR1] Anselin L, Varga A, Acs Z (1997). Local geographic spillovers between university research and high technology innovations. J Urban Econ.

[CR2] Swedish Television (Swe: Sveriges Television) (2020a) Medicine Association Alarms: Several Swedish Somalis among Corona Deaths in the Stockholm Area.

[CR3] Audretsch D, Feldman M (1996). R&D spillovers and the geography of innovation and production. Am Econ Rev.

[CR4] Bi Q, Wu Y, Mei S, Ye C, Zou X, Zhang Z, Liu X, Wei L, Truelove S, Zhang T, Gao W, Cheng C, Tang X, Wu X, Wu Y, Sun B, Huang S, Sun Y, Feng T (2020). Epidemiology and transmission of COVID-19 in 391 cases and 1286 of their close contacts in Shenzhen, China: a retrospective cohort study. Lancet Infect Diseases.

[CR5] Carozzi F, Provenzano S, Roth S (2020) Urban Density and Covid-19, *CEP Discussion Paper No 1711*

[CR6] Chin WCB, Bouffanais R (2020). Spatial super-spreaders and super-susceptibles in human movement networks. Sci Rep.

[CR7] Credit K (2020). Neighbourhood inequity: Exploring the factors underlying racial and ethnic disparities in COVID-19 testing and infection rates using ZIP code data in Chicago and New York. Regional Sci Policy Practice.

[CR8] Drefahl S, Wallace M, Mussino E, Aradhya S, Kolk M, Brandén M, Malmberg B, Andersson G (2020). Socio-demographic risk factors of COVID-19 deaths in Sweden: A nationwide register study. 10.1038/s41467-020-18926-3 PMC7547672 33037218

[CR9] Feldman M (1999). The new economics of innovation, spillovers and agglomeration: a review of empirical studies. Econ Innov New Technol.

[CR10] Florida R (2020) The Coronavirus Class Divide in Cities, Bloomberg CityLab April 7^th^.

[CR11] Florida R, Rodriquez-Pose A, Storper M (2020) Cities in a Post-COVID World, (No. 2041). Utrecht University, Department of Human Geography and Spatial Planning, Group Economic Geography

[CR12] Goodman PS (2020) Sweden has become the world’s cautionary tale, *New York Times*, from July 7^th^,

[CR13] Hägerstrand T (1966). Aspects of the spatial structure of social communication and the diffusion of information. Papers Regional Sci Assoc.

[CR14] Hägerstrand T (1967). Innovation as a spatial process.

[CR15] Hägerstrand T (1970). What about people in regional science?. Papers of the Regional Science Association.

[CR16] Hamidi S, Sabouri S, Ewing R (2020) “Does Density Aggravate the COVID-19 Pandemic? Early Findings and Lessons for Planners,” *Journal of the American Planning Association*, pp 1-15.

[CR17] Health and Social Care Inspectorate (2020a) Presentation from press conference on the 7th of July 2020.

[CR18] Health and Social Care Inspectorate (2020b) IVO deepens the review of care and treatment in special housing for the elderly, Available at: https://www.ivo.se/publicerat-material/nyheter/2020/ivo-fordjupar-granskningen-av-vard-och-behandling-pa-sarskilda-boenden-for-aldre/

[CR19] Johansson B, Klaesson J, Olsson M (2003). Commuters’ non-linear response to time distances. J Geograph Syst.

[CR20] Jolliffe IT (1986). Principal components in regression analysis. In *Principal component analysis* (pp. 129-155). Springer, New York, NY.

[CR21] Karlsten E (2020) Multiple errors in the New York Times article about Sweden’s corona strategy, from July 10^th^.

[CR22] Kuebart A, Stabler M (2020). Infectious diseases as socio-spatial processes: The Covid-19 outbreak in Germany. Tijdschrift Voor Economische En Sociale Geografie.

[CR23] Li W, Zhang B, Lu J, Liu S, Chang Z, Peng C, Liu X, Zhang P, Ling Y, Tao K, Chen J (2020). Characteristics of household transmission of COVID-19. Clin Infect Diseases.

[CR24] Linka K, Peirlinck M, Sahli Costabal F, Kuhl E (2020). Outbreak dynamics of COVID-19 in Europe and the effect of travel restrictions. Comput Methods in Biomech Biomed Eng.

[CR25] Nathan M (2020) The City and the Virus, *Medium*, 14^th^ of May 2020

[CR26] Nguyen LH, Drew DA, Graham MS, Joshi AD, Guo CG, Ma W, Kwon S (2020). Risk of COVID-19 among front-line health-care workers and the general community: a prospective cohort study. Lancet Public Health.

[CR27] Dagens Nyheter (2020) Over-representation of corona-infections in Järvafältet in Stockholm County, from 26^th^ of March 2020

[CR28] Paterlini M (2020) ‘Closing borders is ridiculous’: the epidemiologist behind Sweden’s controversial coronavirus strategy,’ *Nature*, p. 574. 10.1038/d41586-020-01098-x 32317784

[CR29] Porter M (2000) “Locations, clusters and company strategy” In The Oxford handbook of economic geography, Clark and al. eds., Oxford: Oxford U. Press. 253-274

[CR30] The Swedish Public Health Agency (Swe: Folkhälsomyndigheten) (2020) Presence of COVID-19 in different occupational groups

[CR31] Swedish Television (2020b) Serious shortcomings in elderly care – 1,700 homes will be examined.

[CR32] Swedish Television (2020c) Spånga and Kista particularly affected by the coronavirus

[CR33] Viboud C, Bjørnstad ON, Smith DL, Simonsen L, Miller MA, Grenfell BT (2006). Synchrony, waves, and spatial hierarchies in the spread of influenza. *science*, *312*(5772), 447-451. 10.1126/science.1125237 16574822

